# The roles of noninvasive mechanical ventilation with helmet in patients with acute respiratory failure: A systematic review and meta-analysis

**DOI:** 10.1371/journal.pone.0250063

**Published:** 2021-04-15

**Authors:** Shukun Hong, Hongye Wang, Yonggang Tian, Lujun Qiao

**Affiliations:** 1 Department of Intensive Care Unit, Shengli Oilfield Central Hospital, Dongying, China; 2 Department of Obstetrics and Gynecology, Shengli Oilfield Central Hospital, Dongying, China; University of Palermo, ITALY

## Abstract

**Objective:**

To compare the safety and effectiveness between helmet and face mask noninvasive mechanical ventilation (NIMV) in patients with acute respiratory failure (ARF).

**Methods:**

English databases included PubMed, EMBASE, Cochrane Central Register of Controlled Trials and Web of Science. Chinese databases involved Wanfang Data, China Knowledge Resource Integrated Database and Chinese Biological Medicine Database. Randomized controlled trials (RCTs) comparing helmet and face mask NIMV for patients with ARF were searched. Meta-analysis was performed using Review manager 5.1.0.

**Results:**

Twelve trials with a total of 569 patients were eligible. Our meta-analysis showed that, comparing with face mask, helmet could significantly decrease the incidences of intolerance [risk ratio (RR) 0.19; 95% confidence interval (CI) 0.09−0.39], facial skin ulcer (RR 0.19; 95% CI 0.08−0.43) and aerophagia (RR 0.15; 95% CI 0.06−0.37), reduce respiratory rate [mean difference (MD) -3.10; 95% CI -4.85 to -1.34], intubation rate (RR 0.39; 95% CI 0.26−0.59) and hospital mortality (RR 0.62; 95% CI 0.39−0.99) in patients with ARF, and improve oxygenation index in patients with hypoxemic ARF (MD 55.23; 95% CI 31.37−79.09). However, subgroupanalysis for hypercapnic ARF revealed that PaCO_2_ was significantly reduced in face mask group compared with helmet group (MD 5.34; 95% CI 3.41−7.27).

**Conclusion:**

NIMV with helmet can improve the patient’s tolerance, reduce adverse events, increase oxygenation effect, and decrease intubation rate and hospital mortality comparing to face mask. However, the low number of patients from included studies may preclude strong conclusions. Large RCTs are still needed to provide more robust evidence.

## Introduction

Mechanical ventilation, as first-line therapy for acute respiratory failure (ARF) caused by various diseases, can be delivered by invasive and noninvasive methods. Although invasive mechanical ventilation has a better effect on gas exchange and sputum drainage, the complications, such as ventilator-associated pneumonia, airway injury and delirium, occurred more frequently [1–3]. In contrast, noninvasive mechanical ventilation (NIMV), without the use of endotracheal tube, can reduce these complications to a certain extent, with less sedatives use [4]. Numerous clinical studies have demonstrated that patients with exacerbation of chronic obstructive pulmonary disease (COPD) and acute cardiogenic pulmonary edema could benefit from NIMV treatment [5]. On the contrary, the beneficial effects of NIMV in acute hypoxemic respiratory failure remain controversial. Nevertheless, a recent review [6] showed its broad and heterogeneous use in the context of acute hypoxemic respiratory failure due to viral infections and COVID-19, with very few studies specifying the type of interface used.

Traditional interfaces between the patients and noninvasive ventilators include oral, nasal and facial (also named oronasal) masks [7]. Based on a large number of clinical studies, these interfaces involve some shortages, such as patient intolerance, air leakage, facial skin damage caused by compression, etc [8]. These factors often lead to the failure of NIMV therapy and the need for tracheal intubation. Therefore, the traditional interfaces need to be improved. Recently, the helmet, as a new type of interface, has been gradually concerned by clinicians. Yang et al. [9] compared the patients with hypoxemia after aortic dissection who were treated with face mask and helmet NIMV respectively. The results showed that the helmet could effectively improve the patients’ comfort and gas exchange, and reduce complications during the process of NIMV. Randomized controlled trial (RCT) by Patel et al. [10] found that compared with face mask, the helmet could significantly reduce the endotracheal intubation rate and 90-day mortality of patients with acute respiratory distress syndrome (ARDS). However, results from clinical studies were not always consistent. Some researches found that the helmet was not better than the face mask [11], even worse than the latter [12]. Therefore, the current study data brings difficulties to the choice of clinical treatment.

The purpose of our study was to perform a meta-analysis for comparing the safety and effectiveness of helmet NIMV with face mask NIMV in patients with ARF.

## Materials and methods

We performed this study in accordance with the Statement of Preferred Reporting Items for Systematic Reviews and Meta-Analyses [13, 14]. All stages of literature search, study selection, data extraction and quality assessment were done independently by two reviewers. Any discrepancies between the two reviewers were resolved by discussion or arbitration by a third reviewer. No study protocol exists for the systematic review.

### Search strategy

The English electronic databases utilized in our literature search included PubMed, EMBASE, Cochrane Central Register of Controlled Trials and Web of Science. The following Chinese electronic databases were also searched: Wanfang Data, China Knowledge Resource Integrated Database and Chinese Biological Medicine Database. The following search strategy was used in PubMed and changes depending on the rules of each database: (((((((“Respiratory Distress Syndrome, Adult”[Mesh]) OR (“acute respiratory distress syndrome”)) OR (“Pulmonary Disease, Chronic Obstructive”[Mesh])) OR (“chronic obstructive pulmonary disease”)) OR (“Respiratory Insufficiency”[Mesh])) OR (“respiratory failure”)) AND ((((((“Respiration, Artificial”[Mesh]) OR (“mechanical ventilation”)) OR (“Continuous Positive Airway Pressure”[Mesh])) OR (“continuous positive airway pressure”)) OR (“Noninvasive Ventilation”)) OR (“Noninvasive Ventilation”[Mesh]))) AND ((((“facial mask”) OR (“face mask”)) OR (helmet)) OR (“Head Protective Devices”[Mesh])). No language restriction was applied during literature searches. All references cited in the relevant articles were screened to identify additional publications. The latest search was conducted on 30 May 2020.

### Study selection

We evaluated the eligible studies that met all of the inclusion criteria as follows: (1) RCTs; (2) compared the helmet NIMV with face mask NIMV for adult patients with ARF; (3) reported on at least one of the outcomes mentioned below. In cases of duplicates, the most recent or the most complete publication was used. Studies comparing helmet NIMV with oxygen therapy for patients with ARF were excluded. Retrospective studies, reviews, case reports and conference abstracts which presented insufficient information were excluded. To assess chance-corrected agreement between reviewers, Cohen’s kappa statistic was employed (SPSS, version 18.0).

### Data extraction and quality assessment

For each study, the following data were extracted using standardized data extraction forms: the first author’s last name; year of publication; country; study interval; cases and mean age in each group; type of ARF; underlying diseases; ventilator settings; primary outcome of each study; other study features and data needed for quality assessment. The outcomes analyzed in this study were the incidences of intolerance, facial skin ulcer, and aerophagia, respiratory rate, endotracheal intubation rate, oxygenation index, partial pressure of carbon dioxide (PaCO_2_), length of stay in the intensive care unit and hospital mortality. The methodological quality of the included studies was assessed according to the criteria specified by the Cochrane Collaboration [15], and the summary figures of risk of bias were generated. The assessed items of risk of bias involved random sequence generation (selection bias), allocation concealment (selection bias), blinding of participants and personnel (performance bias), blinding of the outcome assessment (detection bias), incomplete outcome data (attrition bias), selective reporting (reporting bias), and other bias.

### Statistical analysis

Analyses were conducted by using the statistical software Review Manager, version 5.1.0 (The Cochrane Collaboration, 2011). As we previously reported [16, 17], for continuous variables, the mean difference (MD) with corresponding 95% confidence interval (CI) was calculated in inverse variance method. For dichotomous variables, the pooled risk ratio (RR) with corresponding 95% CI was aggregated in Mantel–Haenszel method. All results in our analysis were evaluated for clinical and statistical heterogeneity. Clinical heterogeneity was discussed when appropriate and possible. Given the inconsistence of ARF types in this study, subgroup analysis was performed. Statistical heterogeneity was assessed by I^2^ statistic and Cochran’s *Q* test with p<0.1 considered as significant. If the statistical heterogeneity was not significant, the fixed effect model would be used; otherwise, the random effects model would be applied. Forest plot was constructed to graphically assess the statistical heterogeneity by displaying effect estimates and 95% CI for both individual studies and meta-analyses. Publication bias was evaluated by the Egger’s regression with p<0.1 considered as significant (STATA 12.0). Two-sided p values were used throughout.

## Results

### Study selection

**Fig 1:**
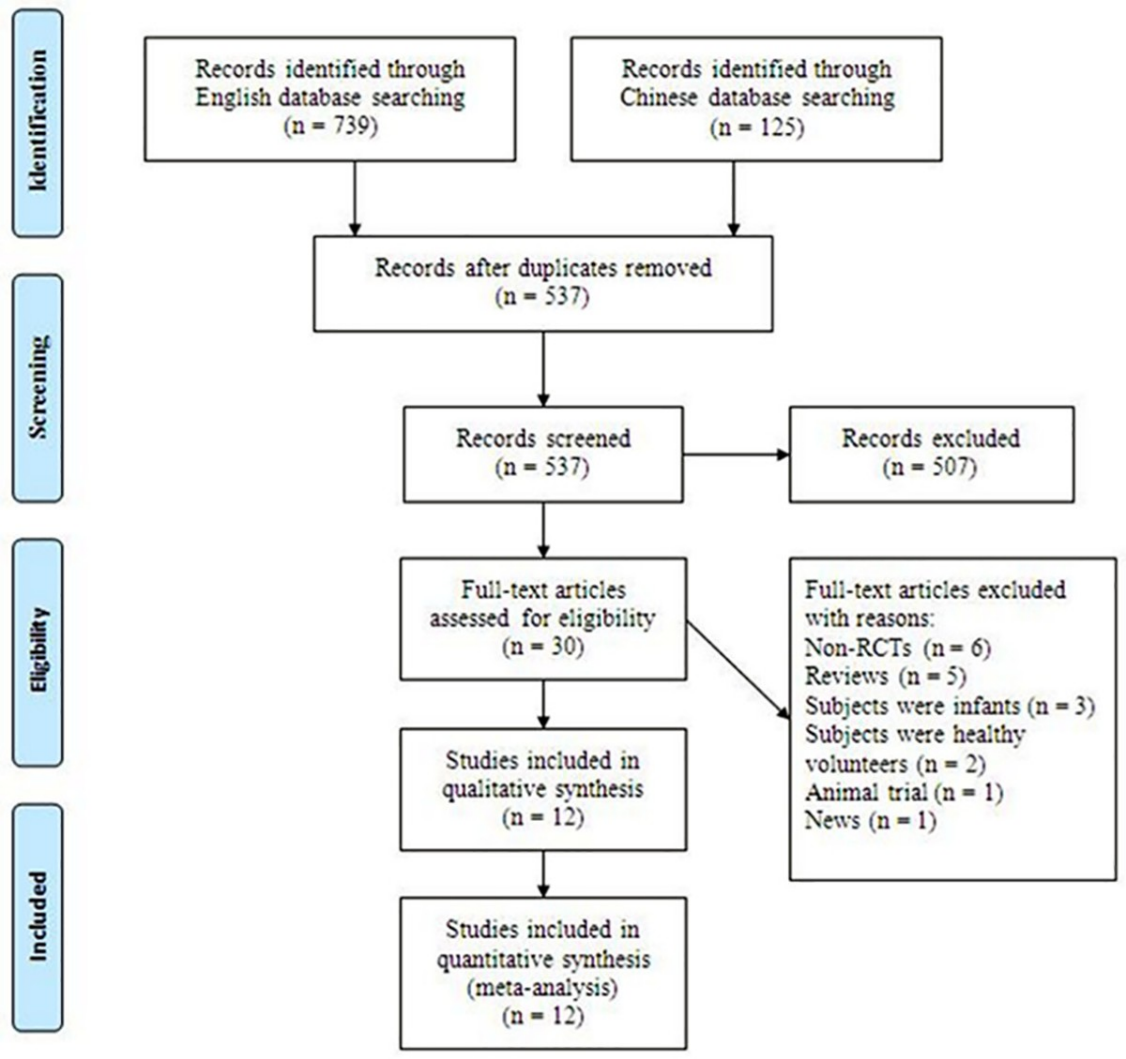
Study flow diagram chart.

A total of 864 citations were identified from literature searches. After titles and abstracts screening, 30 citations with full-text were retrieved for detailed evaluation. After reviewing, 18 studies were excluded for the following reasons: non-RCT (n = 6) [18–23], review article (n = 5) [24–28], subjects were infants (n = 3) [29–31], subjects were healthy volunteers (n = 2) [32, 33], animal trial (n = 1) [34] and news (n = 1) [35]. Finally, seven English studies [9–12, 36–38] and five Chinese studies [39–43] matched the inclusion criteria and were suitable for our meta-analysis. The flow diagram in Fig 1 details the selection process. There was excellent agreement between reviewers for study inclusion (κ = 0.90). A total of 569 subjects were analyzed, of which 288 (50.6%) received helmet NIMV and 281 (49.4%) received face mask NIMV.

### Study characteristics

The characteristics of the twelve eligible articles are summarized in Table 1. Six studies [9, 39–43] were conducted in China, three [12, 37, 38] in Italy, two [11, 36] in Turkey, and one [10] in United States. The study interval in each trial ranged from 2005 to 2018. The mean age of the subjects varied between 45.13 years and 78.48 years across the studies and was not significantly different between the two groups (MD 0.09 years; 95% CI −1.16 to 1.33; p>0.05; I^2^ = 0%). Of 12 included RCTs, seven trials [11, 12, 36–38, 42, 43] enrolled patients with hypercapnic ARF, the others [9, 10, 39–41] employed patients with hypoxemic ARF. As shown in Table 1, ventilator settings were not consistent across the studies.

**Table 1:** The characteristics of the included studies.

Author, year	Study design	Country	Study interval	Sample size		Mean age		Type of ARF	Underlying diseases	Ventilator settings	Primary outcome
				H	FM	H	FM				
Navalesi, 2007	RCT	Italy	NA	5	5	NA		Hypercapnic	AECOPD	Both groups had same settings. PS 12 cmH_2_O; PEEP 5 cmH_2_O; FiO_2_ was set to maintain SpO_2_ at 93%-96%.	Gas exchange, inspiratory effort, patient–ventilator synchrony, comfort
Zhang, 2008	RCT	China	2005–2006	20	20	72	73	Hypercapnic	AECOPD	Both groups had same settings. PEEP 4–6 cmH_2_O; PS was initially set at 6–8 cmH_2_O, then progressively raised in 2–3 cmH_2_O steps until the RR was <25 bpm and accessory muscle activity disappeared; SpO_2_ was maintained at 90%-95%.	Intubation rate, hospital mortality
Zhang, 2010	RCT	China	2005–2006	20	20	NA		Hypoxemic	SCAP, ARDS, cardiogenic pulmonary edema, pulmonary interstitial fibrosis	Both groups had same settings. PEEP 4–6 cmH_2_O; PS was initially set at 6–8 cmH_2_O, then progressively raised in 2–3 cmH_2_O steps until the RR was <25 bpm and accessory muscle activity disappeared; SpO_2_ was maintained at 90%-95%.	Intubation rate, hospital mortality
Ali, 2011	RCT	Turkey	NA	15	15	59.4	58.5	Hypercapnic	AECOPD	Both groups had same settings. PS 10 cmH_2_O; PEEP 5–7 cmH_2_O; FiO_2_ 0.4.	Gas exchange, respiratory rate, hemodynamics, ICU stay, PTS, intubation rate, complications
Antonaglia, 2011	RCT	Italy	2007	20	20	69	71	Hypercapnic	AECOPD	Both groups had same settings. PEEP 5 cmH_2_O; PS was initially set at 15 cmH_2_O, then progressively raised in 2 cmH_2_O steps until the RR was ⩽30 bpm, accessory muscle activity disappeared and the patient was comfortable; FiO_2_ was set to maintain SpO_2_ at >90%.	Gas exchange, intubation rate, ICU stay, complications
Pisani, 2015	RCT	Italy	2012–2014	39	41	78.36	78.48	Hypercapnic	AECOPD	H: PEEP >5 cmH_2_O; PS ⩾16cmH_2_O; other pressure increments were made to keep RR <20 bpm and disappearance of accessory muscle activity.	Gas exchange, PTS
										FM: PEEP 3–5 cmH_2_O; PS was set to obtain a tidal volume of 6–8 mL·kg^-1^ of body weight.	
Yang, 2015	RCT	China	2013–2014	20	20	52.7	55.5	Hypoxemic	Hypoxemia after aortic dissection	H: PEEP 8–10 cmH_2_O; FiO_2_ 0.4–0.5; SpO_2_ was maintained at >95%.	Gas exchange
										FM: PS10-20 cmH_2_O; PEEP0-4 cmH_2_O; FiO_2_ 0.6–1.0.	
Özlem, 2015	RCT	Turkey	2011–2012	25	23	69.5	64.3	Hypercapnic	AECOPD	Both groups had same settings. PEEP 5–7 cmH_2_O; PS was initially set at 10 cmH_2_O, then progressively raised in 2 cmH_2_O steps to obtain a tidal volume of 6–8 mL·kg^-1^ of body weight; FiO_2_ was set to maintain SpO_2_ at >92%.	Gas exchange, respiratory rate, PTS, complications, ICU stay, duration of NIMV, hospital mortality
Patel, 2016	RCT	USA	2012–2015	44	39	58	60.9	Hypoxemic	ARDS	Both groups had same settings. FiO_2_ ⩽0.6; PEEP was increased in increments of 2–3 cmH_2_O to maintain SpO_2_ at >90%; PS was increased in increments of 2–3 cmH_2_O to obtain a RR ⩽25 bpm and disappearance of accessory muscle activity.	Intubation rate
Yang, 2016	RCT	China	2013–2014	25	25	60.5	61.1	Hypoxemic	Hypoxemia after CABG	H: PEEP 8–10 cmH_2_O; FiO_2_ 0.4–0.5; SpO_2_ was maintained at >95%.	Gas exchange
										FM: PS10-12 cmH_2_O; PEEP 0–4 cmH_2_O; FiO_2_ 0.5–0.8.	
Wang, 2017	RCT	China	2011–2015	23	23	55.89	56.12	Hypoxemic	Hypoxemia after CABG	H: PEEP 8–10 cmH_2_O; FiO_2_ 0.4–0.5; SpO_2_ was maintained at >95%.	Gas exchange
										FM: PS 10–12 cmH_2_O; PEEP0-4 cmH_2_O; FiO_2_ 0.5–0.8.	
Ma, 2019	RCT	China	2017–2018	32	30	45.13	44.52	Hypercapnic	AECOPD	NA	Gas exchange

H, helmet group; FM, face mask group, ARF, acute respiratory failure; RCT, randomized controlled trial; NA, not available; AECOPD, acute exacerbation of chronic obstructive pulmonary disease; PS, pressure support; PEEP, positive end-expiratory pressure; FiO_2_, fraction of inspired oxygen; SpO_2_, peripheral oxygen saturation; RR, respiratory rate; bpm, breaths per minute; SCAP, severe community-acquired pneumonia; ARDS, acute respiratory distress syndrome; PTS, patient tolerance scale; NIMV, noninvasive mechanical ventilation; CABG, coronary artery bypass grafting.

### Quality assessment and publication bias

**Fig 2:**
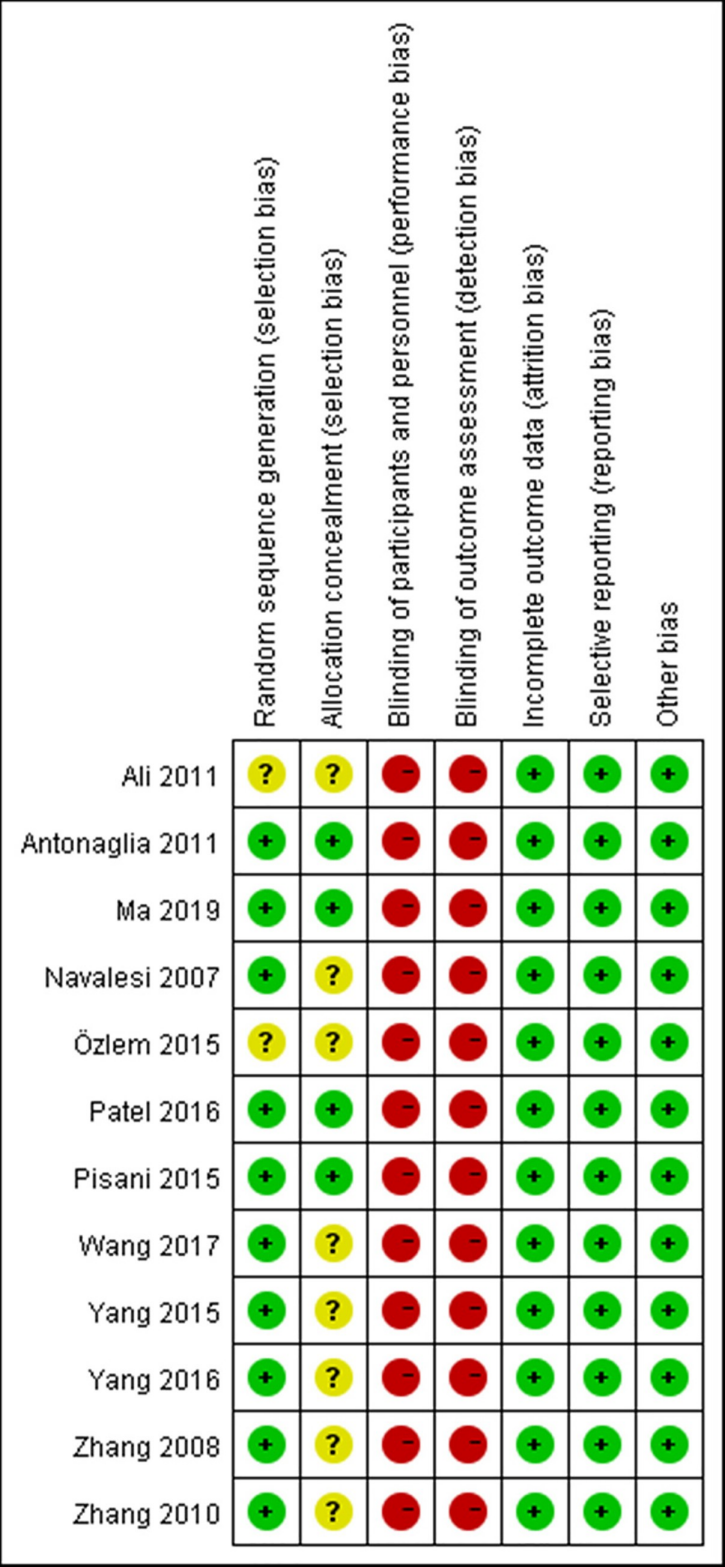
Risk of bias summary.

**Fig 3:**
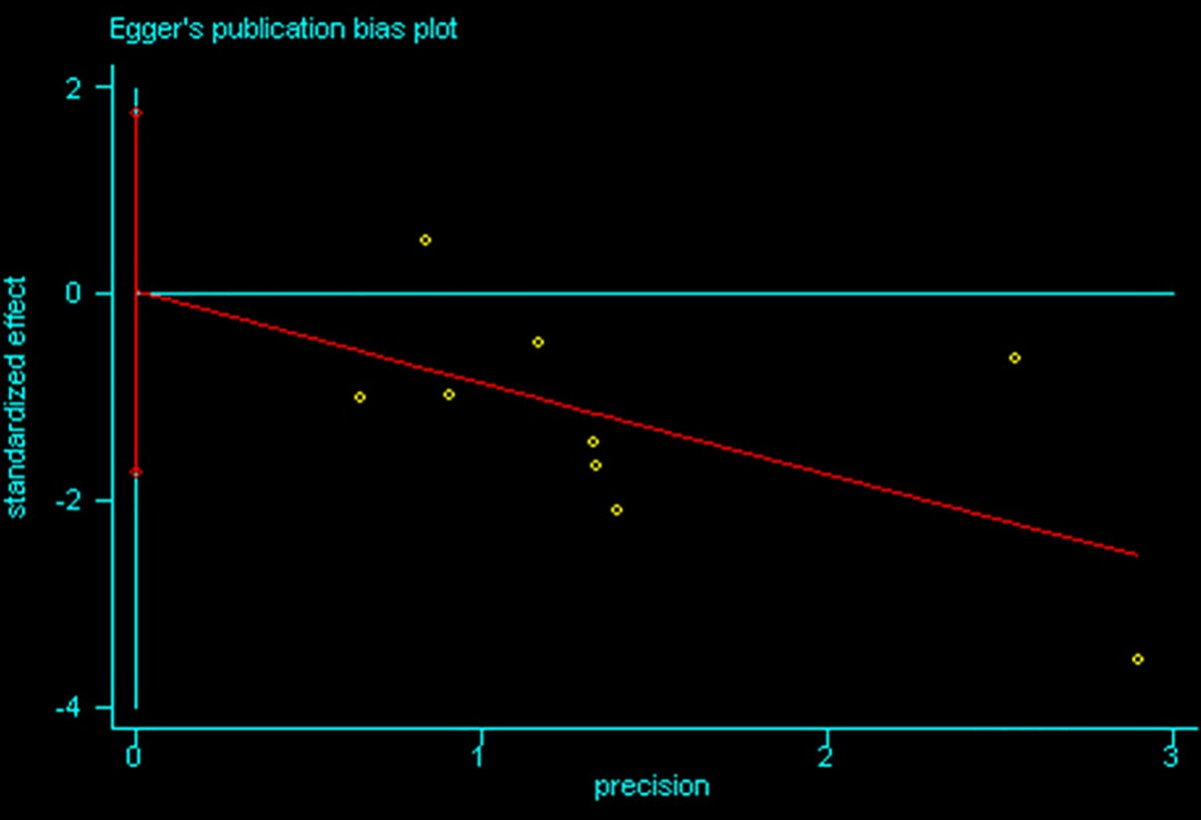
Egger’s regression analysis for publication bias.

The summary of risk of bias assessment is presented in Fig 2. Of 12 included RCTs, no trial was classified as low risk. Ten trials [9, 10, 12, 37–43] reported the appropriate method of randomization; four trials [10, 37, 38, 43] described the allocation concealment in detail. Due to the intrinsic characteristic of study, double blinding was not possible, resulting that all trials were considered as high risk in performance and detection bias. All trials were graded as low risk in terms of incomplete outcome data, selective reporting and other bias. Overall, the included studies were of moderate quality. The Egger’s regression analysis demonstrated that no publication bias was detected (95% CI of intercept -1.72 to 1.74; p>0.1) (Fig 3).

### Quantitative data synthesis

#### Intolerance

**Fig 4:**
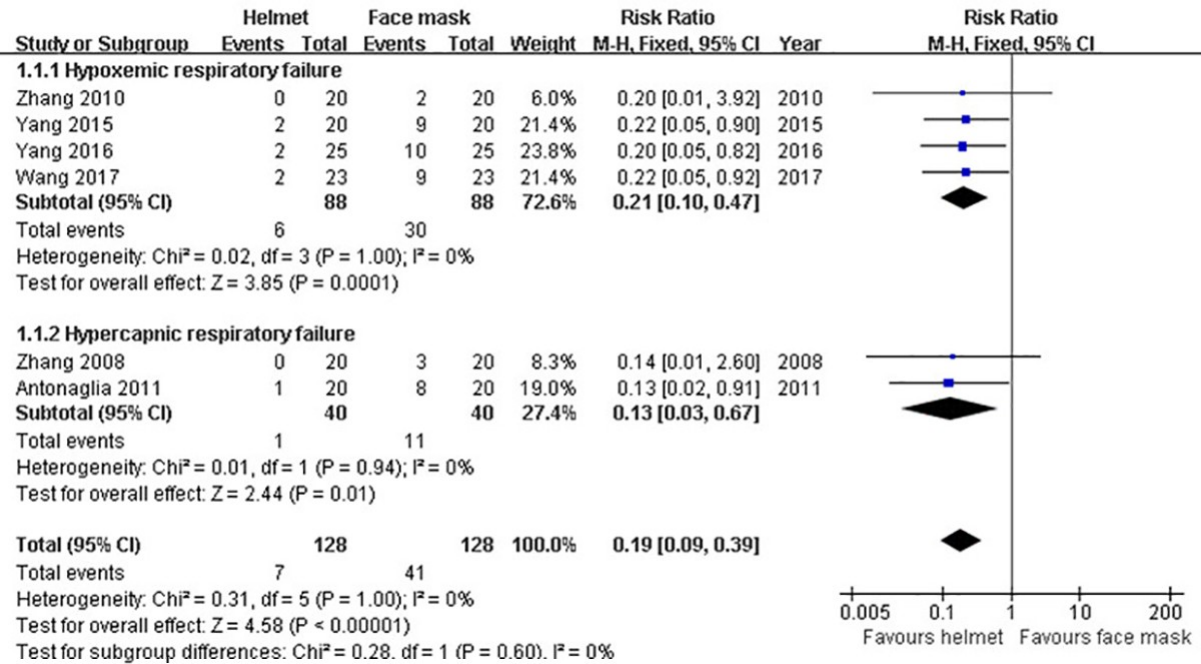
Forest plot of intolerance.

In our analysis, there were six studies [9, 37, 39–42] providing the data of intolerance happened during NIMV. Overall, the rate of intolerance was 5.5% (7/128) in the helmet group and 32% (41/128) in the face mask group, respectively. Due to the non-significant heterogeneity across studies (p>0.1; I^2^ = 0%), fixed-effect model was used. Our meta-analysis showed that the incidence of intolerance was significantly decreased in helmet group compared with face mask group (RR 0.19; 95% CI 0.09−0.39; p<0.001). Moreover, subgroup analysis revealed that this result was unchanged both in subgroups of hypercapnic ARF (RR 0.13; 95% CI 0.03–0.67; p = 0.01) and hypoxemic ARF (RR 0.21; 95% CI 0.10–0.47; p<0.001) (Table 2 and Fig 4).

**Table 2:** Meta-analyses for comparing helmet versus face mask.

Outcome	Studies	Cases	Statistical method	Effect estimate	p for HG	I^2^	p
Intolerance	6	256	RR (M-H, Fixed, 95% CI)	0.19 (0.09−0.39)	1.00	0%	<0.001
Hypercapnic ARF	2	80	RR (M-H, Fixed, 95% CI)	0.13 (0.03−0.67)	0.94	0%	0.01
Hypoxemic ARF	4	176	RR (M-H, Fixed, 95% CI)	0.21 (0.10−0.47)	1.00	0%	<0.001
Facial skin ulcer	7	329	RR (M-H, Fixed, 95% CI)	0.19 (0.08−0.43)	0.50	0%	<0.001
Hypercapnic ARF	2	70	RR (M-H, Fixed, 95% CI)	0.25 (0.03−2.13)	0.82	0%	0.21
Hypoxemic ARF	5	259	RR (M-H, Fixed, 95% CI)	0.18 (0.07−0.44)	0.26	25%	<0.001
Aerophagia	6	296	RR (M-H, Fixed, 95% CI)	0.15 (0.06−0.37)	0.38	6%	<0.001
Hypercapnic ARF	2	120	RR (M-H, Fixed, 95% CI)	0.74 (0.15−3.59)	0.54	0%	0.71
Hypoxemic ARF	4	176	RR (M-H, Fixed, 95% CI)	0.08 (0.02−0.29)	0.94	0%	<0.001
Respiratory rate	9	465	MD (IV, Random, 95% CI)	-3.10 (-4.85 to -1.34)	<0.01	89%	<0.001
Hypercapnic ARF	5	252	MD (IV, Random, 95% CI)	-1.03 (-1.39 to -0.68)	0.64	0%	<0.001
Hypoxemic ARF	4	213	MD (IV, Random, 95% CI)	-5.11 (-6.92 to -3.29)	0.03	66%	<0.001
Intubation rate	9	451	RR (M-H, Fixed, 95% CI)	0.39 (0.26−0.59)	0.56	0%	<0.001
Hypercapnic ARF	5	238	RR (M-H, Fixed, 95% CI)	0.51 (0.28−0.92)	0.38	4%	0.03
Hypoxemic ARF	4	213	RR (M-H, Fixed, 95% CI)	0.33 (0.19−0.56)	0.85	0%	<0.001
Oxygenation index	8	348	MD (IV, Random, 95% CI)	27.76 (9.39−46.13)	<0.01	83%	0.003
Hypercapnic ARF	4	172	MD (IV, Random, 95% CI)	7.20 (-3.10 to 17.50)	0.23	30%	0.17
Hypoxemic ARF	4	176	MD (IV, Random, 95% CI)	55.23 (31.37−79.09)	0.05	61%	<0.001
PaCO_2_	8	318	MD (IV, Random, 95% CI)	1.57 (-1.45 to 4.59)	<0.01	89%	0.31
Hypercapnic ARF	5	182	MD (IV, Random, 95% CI)	5.34 (3.41−7.27)	0.34	11%	<0.001
Hypoxemic ARF	3	136	MD (IV, Random, 95% CI)	-2.32 (-3.43 to -1.21)	0.29	19%	<0.001
ICU stay	7	337	MD (IV, Random, 95% CI)	-0.39 (-2.23 to 1.45)	<0.01	98%	0.67
Hypercapnic ARF	3	118	MD (IV, Random, 95% CI)	0.78 (-1.75 to 3.31)	<0.01	97%	0.54
Hypoxemic ARF	4	219	MD (IV, Random, 95% CI)	-1.28 (-2.51 to -0.05)	<0.01	86%	0.04
Hospital mortality	8	403	RR (M-H, Fixed, 95% CI)	0.62 (0.39−0.99)	0.99	0%	0.04
Hypercapnic ARF	4	190	RR (M-H, Fixed, 95% CI)	0.86 (0.36−2.06)	0.99	0%	0.74
Hypoxemic ARF	4	213	RR (M-H, Fixed, 95% CI)	0.54 (0.31−0.93)	0.99	0%	0.03

HG, heterogeneity; RR, risk ratio; M-H, Mantel-Haenszel; Fixed, fixed effect model; CI, confidence interval; ARF, acute respiratory failure; MD, mean difference; IV, inverse variance; Random, random effects model.

#### Facial skin ulcer

**Fig 5:**
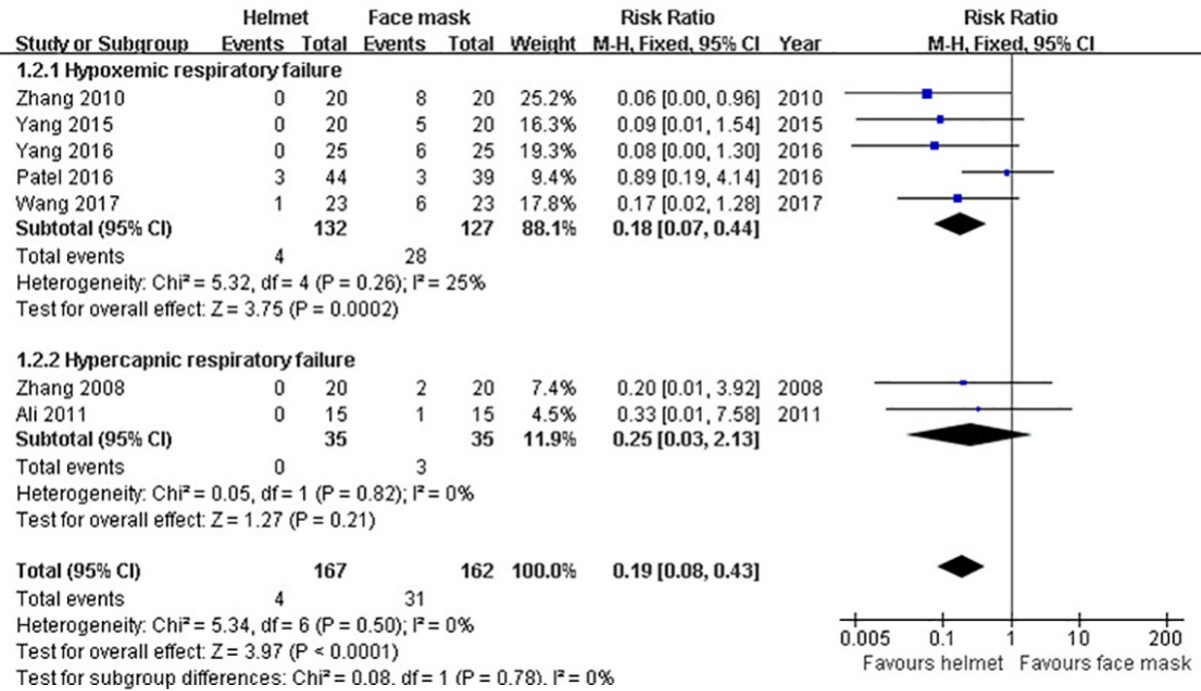
Forest plot of facial skin ulcer.

Seven studies [9, 10, 36, 39–42] reported the information regarding facial skin ulcer after applying NIMV. When seven studies were pooled, 4 (2.4%) patients receiving helmet NIMV and 31 (19.1%) patients receiving face mask NIMV experienced facial skin ulcer. Fixed-effect model was used for data synthesis due to the non-significant heterogeneity across studies (p>0.1; I^2^ = 0%). Our pooling results revealed that the helmet group had less facial skin ulcer rate than the face mask group (RR 0.19; 95% CI 0.08−0.43; p<0.001). However, subgroup analysis found that this significant difference between the groups in facial skin ulcer rate was only remained in subgroup of hypoxemic ARF (RR 0.18; 95% CI 0.07−0.44; p<0.001) rather than hypercapnic ARF (RR 0.25; 95% CI 0.03−2.13; p>0.05) (Table 2 and Fig 5).

#### Aerophagia

**Fig 6:**
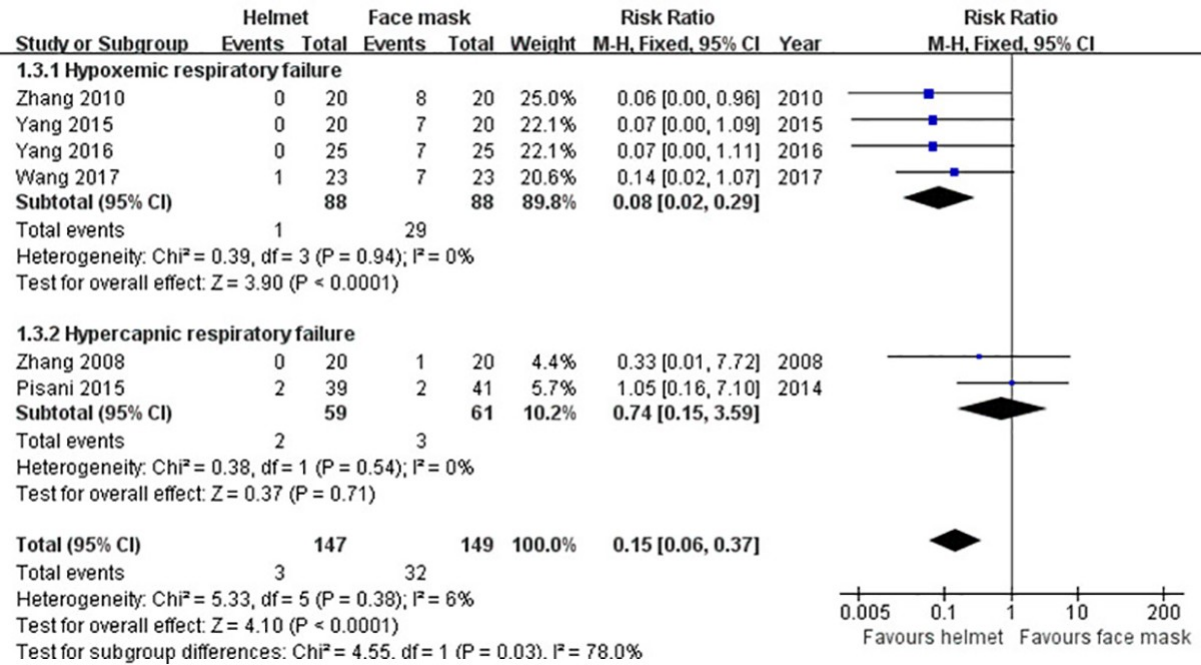
Forest plot of aerophagia.

Data of aerophagia developed during NIMV were described in six trials [9, 38–42]. After pooling data, the incidence of aerophagia in helmet group was reduced by 19.5% compared with face mask group. Meta-analysis on fixed-effect model demonstrated that the difference was statistically significant (RR 0.15; 95% CI 0.06−0.37; p<0.001), which was in accordance with the result of subgroup analysis for hypoxemic ARF (RR 0.08; 95% CI 0.02−0.29; p<0.001). Nevertheless, no significant difference was observed by subgroup analysis for hypercapnic ARF (RR 0.74; 95% CI 0.15−3.59; p>0.05) (Table 2 and Fig 6).

#### Respiratory rate

**Fig 7:**
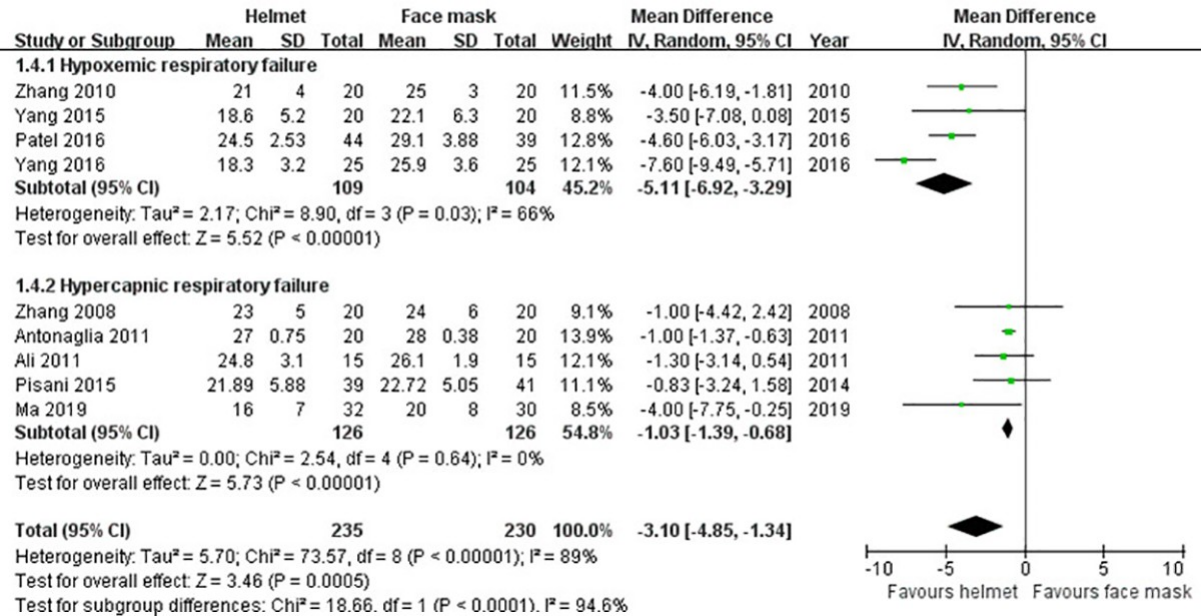
Forest plot of respiratory rate.

Nine included studies [9, 10, 36–39, 41–43] reported the data of respiratory rate. Random effects model was applied owing to a significant heterogeneity across studies (p<0.001; I^2^ = 89%). Our meta-analysis revealed that the difference in respiratory rate between the groups had achieved statistical significance (MD -3.10; 95% CI -4.85 to -1.34; p<0.001). The finding was in line with the results of subgroup analysis for hypercapnic and hypoxemic ARF (Table 2 and Fig 7).

#### Endotracheal intubation

**Fig 8:**
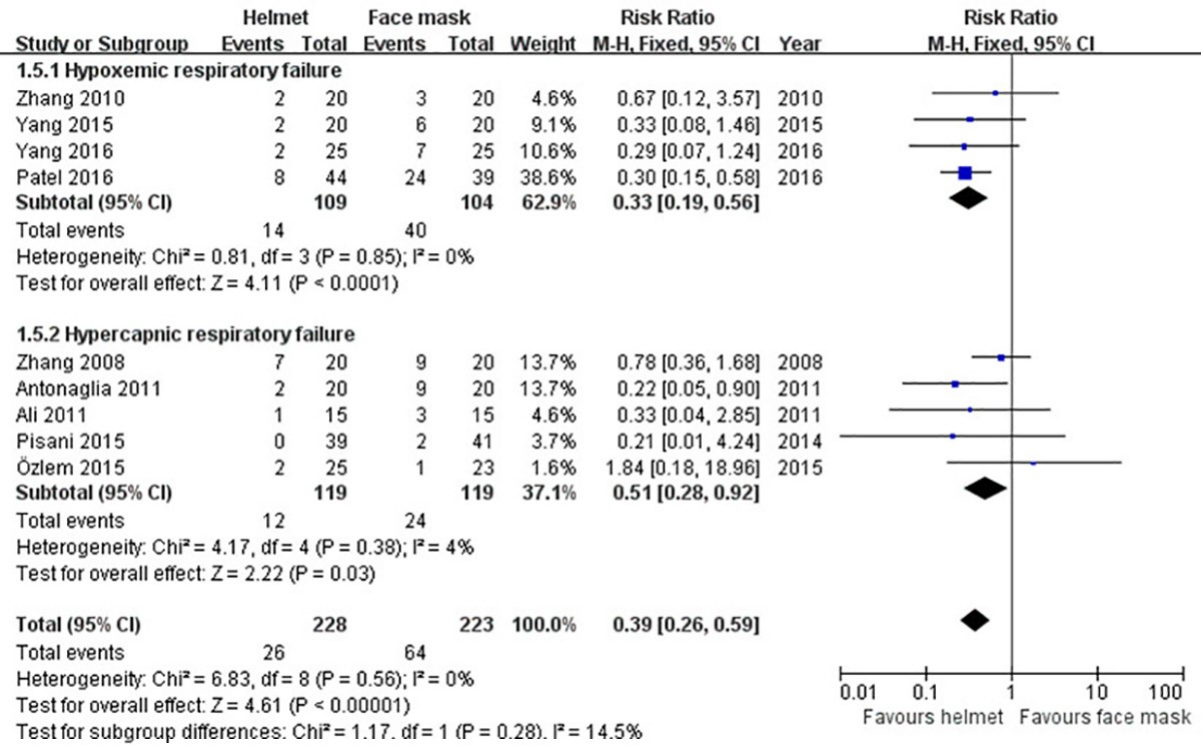
Forest plot of endotracheal intubation.

Data regarding endotracheal intubation was collected from nine studies [9–11, 36–39, 41, 42]. As a whole, 11.4% (26/228) patients experienced endotracheal intubation in helmet group and 28.7% (64/223) in face mask group. As statistical heterogeneity across studies was non-significant (p>0.1; I^2^ = 0%), fixed-effect model was adopted. The pooling results of our meta-analysis (RR 0.39; 95% CI 0.26−0.59; p<0.001) and subgroup analysis (hypercapnic ARF (RR 0.51; 95% CI 0.28−0.92; p<0.05) and hypoxemic ARF (RR 0.33; 95% CI 0.19−0.56; p<0.001)) all showed that the difference in intubation rate between the groups was statistically significant (Table 2 and Fig 8).

#### Oxygenation index

**Fig 9:**
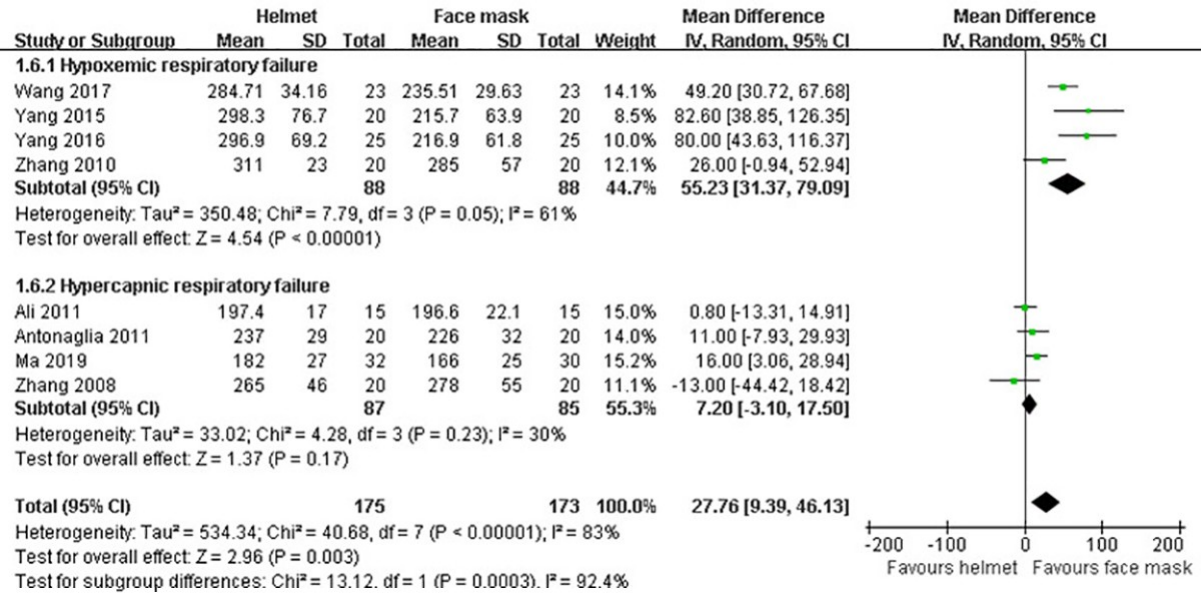
Forest plot of oxygenation index.

There were eight studies [9, 36, 37, 39–43] reporting the information about oxygenation index. Due to the significant heterogeneity among studies (p<0.001; I^2^ = 83%), random effects model was employed. Our meta-analysis (MD 27.76; 95% CI 9.39−46.13; p<0.01) and subgroup analysis for hypoxemic ARF (MD 55.23; 95% CI 31.37−79.09; p<0.001) all revealed that the oxygenation index was increased in helmet group compared with face mask group. However, subgroup analysis for hypercapnic ARF failed to show a significant difference between the groups in oxygenation index (MD 7.20; 95% CI -3.10 to 17.50; p>0.05) (Table 2 and Fig 9).

#### PaCO_2_

**Fig 10:**
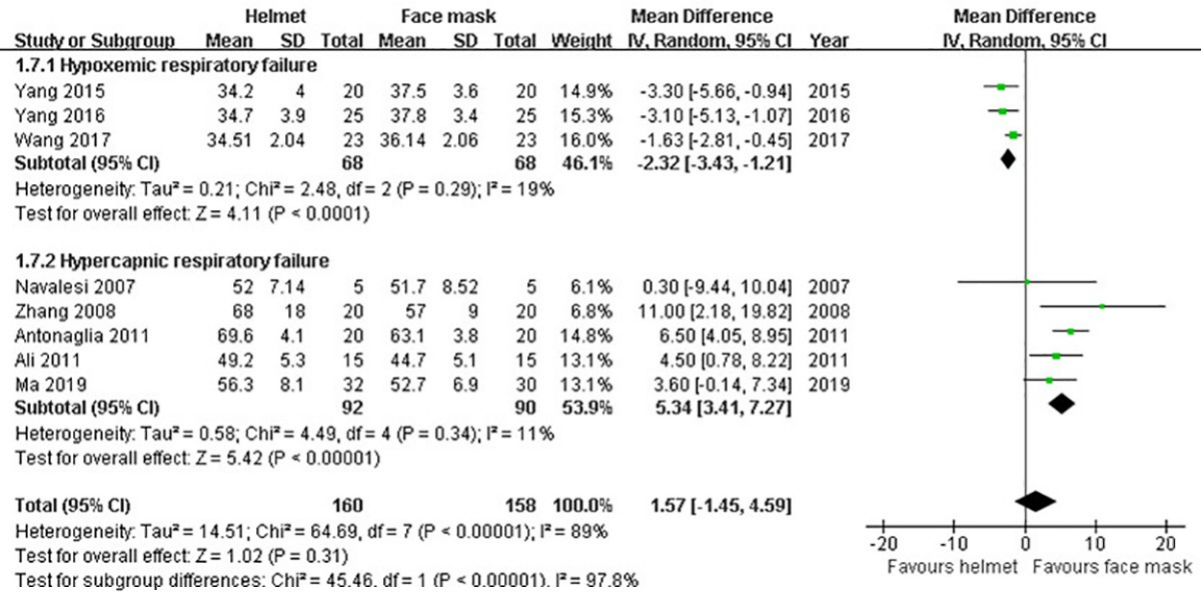
Forest plot of PaCO_2_.

Eight trials in this analysis provided the data of PaCO_2_ after applying NIMV [9, 12, 36, 37, 40–43]. Random effects model was used to synthetize the data because of a significant heterogeneity among studies (p<0.001; I^2^ = 89%). No significant difference between the groups in PaCO_2_ was found in our meta-analysis (MD 1.57; 95% CI -1.45 to 4.59; p>0.05). Subgroup analysis for hypercapnic ARF revealed that PaCO_2_ was significantly reduced in face mask group compared with helmet group (MD 5.34; 95% CI 3.41−7.27; p<0.001). Whereas PaCO_2_ in helmet group was significantly decreased when subgroup analysis for hypoxemic ARF was performed (MD -2.32; 95% CI -3.43 to -1.21; p<0.001) (Table 2 and Fig 10).

#### ICU stay

**Fig 11:**
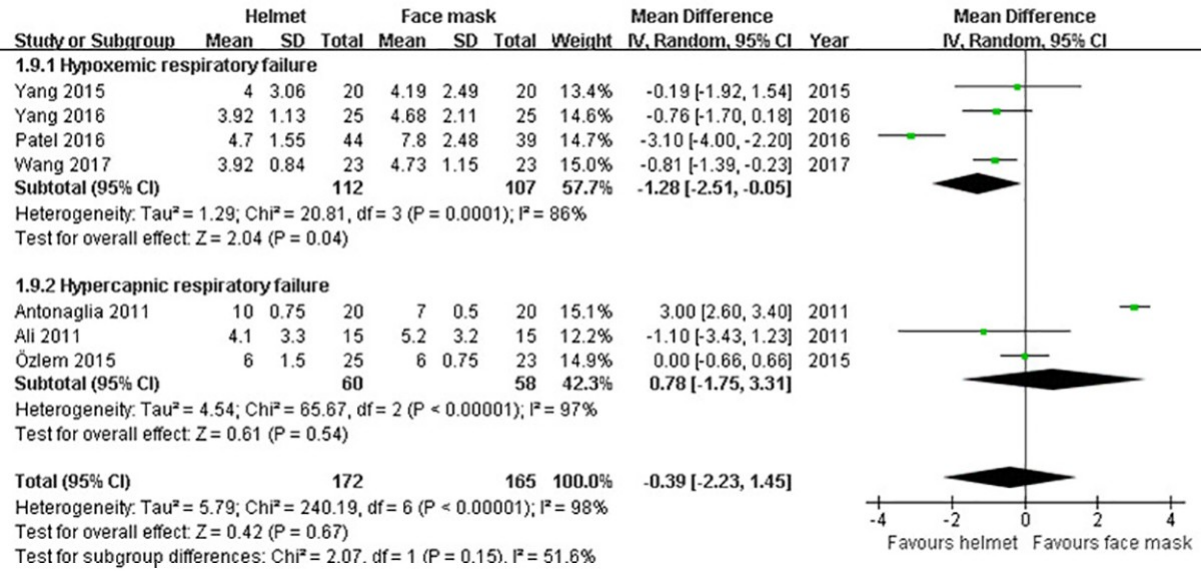
Forest plot of ICU stay.

Information about ICU stay was described in seven trials [9–11, 36, 37, 40, 41]. Owing to a significant heterogeneity across studies (p<0.001; I^2^ = 98%), random effects model was applied. No significant difference in ICU stay between two groups was detected in our meta-analysis (MD -0.39; 95% CI -2.23 to 1.45; p>0.05), or subgroup analysis for hypercapnic ARF (MD 0.78; 95% CI -1.75 to 3.31; p>0.05). However, subgroup analysis for hypoxemic ARF showed a significantly shorter ICU stay in helmet group (MD -1.28; 95% CI -2.51 to -0.05; p<0.05) (Table 2 and Fig 11).

#### Hospital mortality

**Fig 12:**
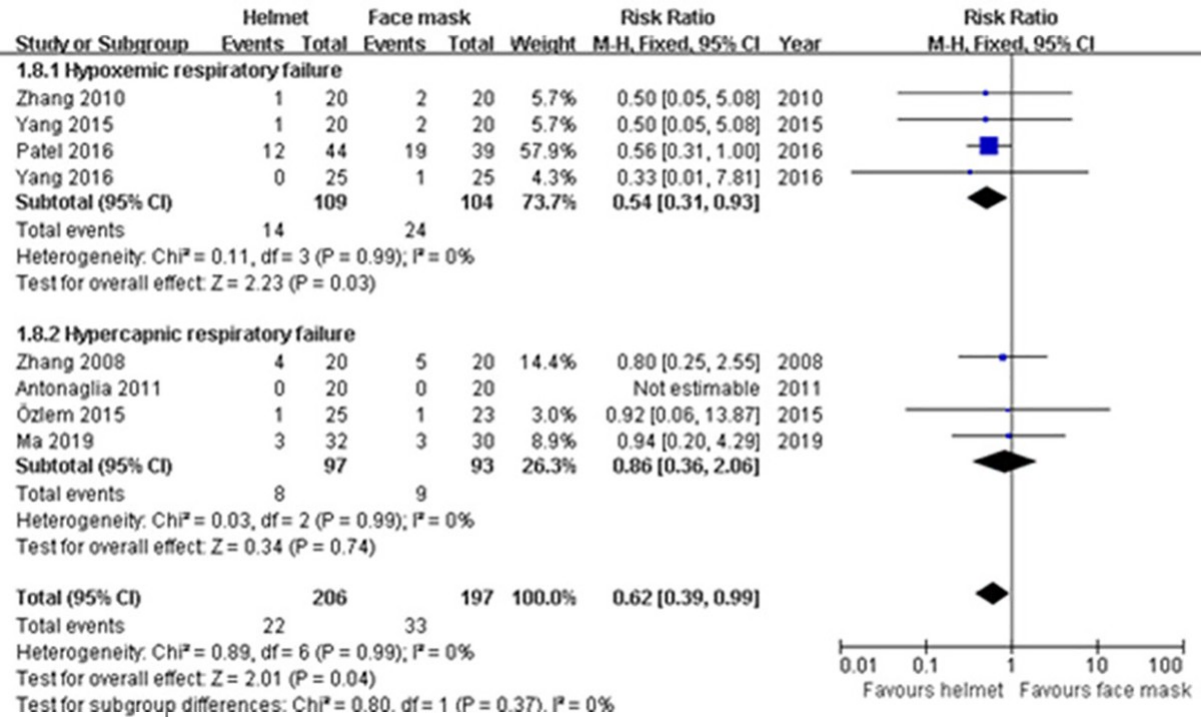
Forest plot of hospital mortality.

There were eight studies reporting the information of hospital mortality [9–11, 37, 39, 41–43]. Overall, the mortality in helmet group and face mask group was 10.7% and 16.8%, respectively. Fixed-effect model was used due to the non-significant heterogeneity across studies (p>0.1; I^2^ = 0%). A significant difference between groups in hospital mortality was observed in our meta-analysis (RR 0.62; 95% CI 0.39−0.99; p<0.05), as well as subgroup analysis for hypoxemic ARF (RR 0.54; 95% CI 0.31−0.93; p<0.05). The difference was not found in subgroup analysis for hypercapnic ARF (RR 0.86; 95% CI 0.36−2.06; p>0.05) (Table 2 and Fig 12).

## Discussion

Our meta-analysis of 12 RCTs showed that the helmet NIMV was associated with better tolerance, less adverse events, and reduced respiratory rate, intubation rate and hospital mortality when compared with the face mask NIMV. Moreover, the helmet NIMV could improve gas exchange in patients with hypoxemic ARF.

In recent 20 years of clinical practice, NIMV has been widely applied, especially for patients with cardiogenic pulmonary edema and COPD [27]. However, NIMV is often interrupted due to the patient intolerance, resulting in treatment failure. The reason may be related to the patient-ventilator interaction of NIMV [44]. The classic manner of interaction is the facial (also named oronasal) mask interface, which usually cause some complications, such as facial skin ulcer, eye irritation, aerophagia, etc., leading to patient intolerance. Compared with the face mask, the helmet can be suitable for patients with different facial shapes, and the patients can contact and communicate with the surrounding environment during ventilation process. A trial by Antonaglia et al. [37] found that the incidence of intolerance in patients with exacerbation of COPD receiving helmet NIMV was significantly less than that in patients receiving face mask NIMV (5% vs. 40%). In a multicenter RCT, Chidini et al. [45] treated infants with ARF caused by respiratory syncytial virus with helmet and face mask NIMV. The results showed that the intolerance rate and trial failure rate in the helmet group were significantly lower than those in the face mask group. Yang et al. [41] recruited patients with hypoxemia after coronary artery bypass grafting surgery to randomly receive NIMV either with the helmet or the face mask. The research team observed that patients treating with helmet NIMV experienced less intolerance, facial skin ulcer and aerophagia. However, in patients with exacerbation of COPD, Pisani et al. [38] found that there was no difference between the two groups in the score of discomfort and incidence of adverse events after ventilation treatment. The results of our study confirmed that the tolerance of helmet NIMV is better than that of face mask NIMV, no matter what type of ARF. Unfortunately, the advantages of helmet NIMV in reducing the incidences of facial skin ulcer and aerophagia were only shown in hypoxemic ARF, but not in hypercapnic ARF. The reason for this difference may be due to the relatively small sample size in the study of hypercapnic ARF. Increasing the sample size may display positive outcomes.

It is well known that avoiding tracheal intubation can reduce the incidence of ventilator-associated pneumonia, and cut down the use of analgesic and sedative drugs. Antonaglia et al. [37] observed that NIMV with helmet can significantly reduce the rate of tracheal intubation compared with NIMV with face mask in patients with exacerbation of COPD. Further analysis indicated that in the face mask group, 88% of the patients with endotracheal intubation failed in NIMV merely due to intolerance. In our study, the respiratory rate of patients treated with helmet NIMV was significantly decreased, reflecting that the respiratory distress was further alleviated. We believe that the decreases of respiratory rate and tracheal intubation rate are closely related to the better tolerance of helmet NIMV.

Compared with oxygen therapy, NIMV can provide certain inspiratory pressure and positive end expiratory pressure (PEEP), so as to increase the minute ventilation volume of the lung, prevent alveolar collapse, reduce intrapulmonary shunt, and improve the ventilation / perfusion ratio, thus promoting gas exchange. Zhang et al. [39] found that helmet NIMV can significantly increase the oxygenation index of patients with hypoxemic ARF compared with face mask NIMV. Yang et al. [9, 41] also reported similar results. The results of our meta-analysis are consistent with the above studies. It is speculated that the reason why helmet NIMV can increase oxygenation index may be related to the better airtightness of the ventilation system and more effective transmission of PEEP. It should be noted that a recently published meta-analysis [26] showed that there was no difference in the effects of two NIMV modes on oxygenation. Through comparative observation, it was found that the meta-analysis simultaneously included RCTs and non-RCTs, and in the analysis of RCTs, it selected two articles that were excluded in our study (the reason for exclusion was that NIMV parameters were not provided, therefore it was not suitable to conduct meta-analysis based on the results). This may be the reason why the above results are inconsistent.

Some studies [36, 37, 42] reported that helmet NIMV was less effective than face mask NIMV in reducing CO_2_ retention in patients with COPD. Similar results were found in our meta-analysis. These findings could possibly be explained by three factors: (1) CO_2_ rebreathing, (2) an increase in ventilation dead space, and (3) less reduction of inspiratory effort. However, it is believed that the fresh gas flow rate of the helmet NIMV can reach 100 ~ 200L / min, which can reduce the risk of CO_2_ rebreathing in the helmet [46]. A study by Antonelli et al. [47] reported that CO_2_ rebreathing with the helmet and the mask in healthy volunteers was similar and always less than 1.5%.

It is considered that due to the soft collar of the helmet, the inspiratory pressure was dissipated partly, resulting in a less efficient reduction of the inspiratory effort. Under this circumstance, the pressurization rate might be lower and sometimes may affect the trigger and cycling, leading to patient–ventilator dyssynchrony [47]. However, it is thought that to overcome the same inspiratory resistance, the pressure required for helmet NIMV is 33% higher than that for face mask NIMV [18]. In addition, Pisani et al. [38] carried out a trial in which the pressure in the helmet group was increased by 30% compared with that in the face mask group. As a result, there was no difference in PaCO_2_ between the two groups. Nevertheless, in the five studies [11, 12, 36, 37, 42] focusing on COPD included in our meta-analysis, the pressure support levels of the two groups were approximately parallel. This may explain the results of our study. Therefore, by increasing the inspiratory pressure, NIMV with helmet may completely achieve the CO_2_ removal level of face mask NIMV, which needs further confirmation by randomized trials.

Our study found that helmet NIMV could reduce the hospital mortality of patients with hypoxemic ARF, which is consistent with the result of a recent study by Xu et al. [27]. Moreover, the follow-up study by Patel et al. [48] demonstrated that the one-year mortality of patients with ARDS could be reduced by helmet NIMV compared with that of face mask NIMV. Regrettably, our study failed to show that the hospital mortality of patients with hypercapnic ARF in helmet NIMV group was decreased. The reason for this difference is not clear. It may be related to the fact that most of the primary causes of hypoxemic ARF are reversible, while most of the primary diseases leading to hypercapnic ARF are irreversible. In addition, some researchers conducted economic analysis [49] showed that the cost of ICU and hospitalization of the helmet NIMV group was significantly lower than that of the face mask NIMV group, reflecting considerable economic advantage.

The results of our study are similar to a previous meta-analysis published by Liu et al [25], but there are some differences. Firstly, In addition to the English databases that were searched by Liu et al, we also retrieved three main Chinese databases. We realized that more comprehensive literature search could reduce publication bias as much as possible. Secondly, we updated the included literature. In the previous study, five case-control studies and six RCTs were eligible and analyzed. The control group of one RCT was venturi oxygen therapy, not face mask NIMV. In contrast, a total of 12 studies included in our meta-analysis were all RCT with homogeneous treatment group and control group, which may be the main strength of our study. We believed that a larger sample size would make the results of our meta-analysis more reliable.

There are some limitations in our analysis, which deserve discussion. First, we observed considerable heterogeneity between the analyzed studies. Clinical heterogeneity among studies principally involves the primary diseases leading to ARF, the inclusion and exclusion criteria of each study, the modes and settings of mechanical ventilation and the definitions of outcomes. Statistical heterogeneity is generally a consequence of these clinical diversities. Although these variations might have influenced the results of our study, we did use a random effects model (in which each study is regarded as estimating a different effect) for data combining when the statistical heterogeneity was significant. Second, most of the included studies referred to data collected almost 10 years ago. Technological advancement might have improved both face mask and helmet NIMV performance, and new literature insights might have changed the way NIMV is set in clinical practice. Third, all the included studies are characterized by a small sample size, single-center design, and mainly run by clinical experts in the field of NIMV and especially helmet NIMV. Thus, the results of our study should be interpreted with caution. Large RCTs are still needed to provide more robust evidence.

## Conclusion

In summary, this meta-analysis showed that compared to face mask NIMV treating patients with ARF, the helmet NIMV could improve the patient’s tolerance, reduce the incidence of complications, and decrease the respiratory rate, tracheal intubation rate and hospital mortality. Moreover, the oxygenation index of patients with hypoxemic ARF could be increased by NIMV with helmet. Increasing inspiratory pressure may make up for the deficiency of the helmet NIMV in the removal of CO_2_. In view of the possibility that the low number of patients from included studies may preclude strong conclusions, large RCTs are still needed to provide more robust evidence.

## Supporting information

S1 Checklist PRISMA checklist. (DOC)

## References

[1] Pettenuzzo T, Fan E. 2016 Year in Review: Mechanical Ventilation. Respir Care. 2017;62(5):629–35. 10.4187/respcare.05545 28442589

[2] Miske LJ, Stetzer M, Garcia M, Stellar JJ. Airways and Injuries: Protecting Our Pediatric Patients from Respiratory Device-Related Pressure Injuries. Crit Care Nurs Clin North Am. 2017;29(2):187–204. 10.1016/j.cnc.2017.01.006 28460700

[3] Cooper JD. Tracheal Injuries Complicating Prolonged Intubation and Tracheostomy. Thorac Surg Clin. 2018;28(2):139–44. 10.1016/j.thorsurg.2018.01.001 29627046

[4] Hess DR. Noninvasive ventilation for acute respiratory failure. Respir Care. 2013;58(6):950–72. 10.4187/respcare.02319 23709194

[5] Joshi N, Estes MK, Shipley K, Lee HD. Noninvasive Ventilation For Patients In Acute Respiratory Distress: An Update. Emerg Med Pract. 2017;19(2):1–20. 28118145

[6] Crimi C, Noto A, Cortegiani A, Impellizzeri P, Elliott M, Ambrosino N, et al. Noninvasive respiratory support in acute hypoxemic respiratory failure associated with COVID-19 and other viral infections. Minerva Anestesiol. 2020;86(11):1190–204. 10.23736/S0375-9393.20.14785-0 32756535

[7] Ma Z, Drinnan M, Hyde P, Munguia J. Mask interface for continuous positive airway pressure therapy: selection and design considerations. Expert Rev Med Devices. 2018;15(10):725–33. 10.1080/17434440.2018.1525291 30227754

[8] Carron M, Freo U, BaHammam AS, Dellweg D, Guarracino F, Cosentini R, et al. Complications of non-invasive ventilation techniques: a comprehensive qualitative review of randomized trials. Br J Anaesth. 2013;110(6):896–914. 10.1093/bja/aet070 23562934

[9] Yang Y, Sun L, Liu N, Hou X, Wang H, Jia M. Effects of Noninvasive Positive-Pressure Ventilation with Different Interfaces in Patients with Hypoxemia after Surgery for Stanford Type A Aortic Dissection. Med Sci Monit. 2015;21:2294–304. 10.12659/MSM.893956 26250834 PMC4532218

[10] Patel BK, Wolfe KS, Pohlman AS, Hall JB, Kress JP. Effect of Noninvasive Ventilation Delivered by Helmet vs Face Mask on the Rate of Endotracheal Intubation in Patients With Acute Respiratory Distress Syndrome: A Randomized Clinical Trial. JAMA. 2016;315(22):2435–41. 10.1001/jama.2016.6338 27179847 PMC4967560

[11] Ozlem CG, Ali A, Fatma U, Mehtap T, Saziye S. Comparison of helmet and facial mask during noninvasive ventilation in patients with acute exacerbation of chronic obstructive pulmonary disease: a randomized controlled study. Turk J Med Sci. 2015;45(3):600–6. 10.3906/sag-1401-109 26281326

[12] Navalesi P, Costa R, Ceriana P, Carlucci A, Prinianakis G, Antonelli M, et al. Non-invasive ventilation in chronic obstructive pulmonary disease patients: helmet versus facial mask. Intensive Care Med. 2007;33(1):74–81. 10.1007/s00134-006-0391-3 17039354

[13] Knobloch K, Yoon U, Vogt PM. Preferred reporting items for systematic reviews and meta-analyses (PRISMA) statement and publication bias. J Craniomaxillofac Surg. 2011;39(2):91–2. 10.1016/j.jcms.2010.11.001 21145753

[14] Page MJ, Moher D. Evaluations of the uptake and impact of the Preferred Reporting Items for Systematic reviews and Meta-Analyses (PRISMA) Statement and extensions: a scoping review. Syst Rev. 2017;6(1):263. 10.1186/s13643-017-0663-8 29258593 PMC5738221

[15] Higgins JPT, Green S. Cochrane Handbook for Systematic Reviews of Interventions Version 5.1.0 [updated March 2011]. The Cochrane Collaboration, 2011. Available from http://handbook.cochrane.org.

[16] Wang H, Hong S, Liu Y, Duan Y, Yin H. High inspired oxygen versus low inspired oxygen for reducing surgical site infection: a meta-analysis. Int Wound J. 2017;14(1):46–52. 10.1111/iwj.12548 26695819 PMC7949680

[17] Wang H, Hong S, Teng H, Qiao L, Yin H. Subcuticular sutures versus staples for skin closure after cesarean delivery: a meta-analysis. J Matern Fetal Neonatal Med. 2016;29(22):3705–11. 10.3109/14767058.2016.1141886 26785886

[18] Vargas F, Thille A, Lyazidi A, Campo FR, Brochard L. Helmet with specific settings versus facemask for noninvasive ventilation. Crit Care Med. 2009;37(6):1921–8. 10.1097/CCM.0b013e31819fff93 19384209

[19] Rodriguez GL, Medina A, Modesto IAV, Palacios LM, Mayordomo-Colunga J, Vivanco-Allende A, et al. Safety of aerosol therapy in children during noninvasive ventilation with helmet and total face mask. Med Intensiva. 2019;43(8):474–9. 10.1016/j.medin.2018.06.003 30060892

[20] Tonnelier JM, Prat G, Nowak E, Goetghebeur D, Renault A, Boles JM, et al. Noninvasive continuous positive airway pressure ventilation using a new helmet interface: a case-control prospective pilot study. Intensive Care Med. 2003;29(11):2077–80. 10.1007/s00134-003-1925-6 14669764

[21] Principi T, Pantanetti S, Catani F, Elisei D, Gabbanelli V, Pelaia P, et al. Noninvasive continuous positive airway pressure delivered by helmet in hematological malignancy patients with hypoxemic acute respiratory failure. Intensive Care Med. 2004;30(1):147–50. 10.1007/s00134-003-2056-9 14593457

[22] Antonelli M, Conti G, Pelosi P, Gregoretti C, Pennisi MA, Costa R, et al. New treatment of acute hypoxemic respiratory failure: noninvasive pressure support ventilation delivered by helmet—a pilot controlled trial. Crit Care Med. 2002;30(3):602–8. 10.1097/00003246-200203000-00019 11990923

[23] Antonelli M, Pennisi MA, Pelosi P, Gregoretti C, Squadrone V, Rocco M, et al. Noninvasive positive pressure ventilation using a helmet in patients with acute exacerbation of chronic obstructive pulmonary disease: a feasibility study. Anesthesiology. 2004;100(1):16–24. 10.1097/00000542-200401000-00007 14695719

[24] Esquinas RA, Papadakos PJ, Carron M, Cosentini R, Chiumello D. Clinical review: Helmet and non-invasive mechanical ventilation in critically ill patients. Crit Care. 2013;17(2):223. 10.1186/cc11875 23680299 PMC3672531

[25] Liu Q, Gao Y, Chen R, Cheng Z. Noninvasive ventilation with helmet versus control strategy in patients with acute respiratory failure: a systematic review and meta-analysis of controlled studies. Crit Care. 2016;20:265. 10.1186/s13054-016-1449-4 27549178 PMC4994276

[26] Li J, Zeng M, Liang Z. Efficacy of ventilation with a helmet versus face mask in patients with acute respiratory failure: a meta-analysis. Chinese Journal of Respiratory and Critical Care Medicine. 2017(2):165–74.

[27] Xu XP, Zhang XC, Hu SL, Xu JY, Xie JF, Liu SQ, et al. Noninvasive Ventilation in Acute Hypoxemic Nonhypercapnic Respiratory Failure: A Systematic Review and Meta-Analysis. Crit Care Med. 2017;45(7):e727–33. 10.1097/CCM.0000000000002361 28441237 PMC5470860

[28] Zheng X, Qu NN, Wang WP, Shu P, Shi XL, Deng H, et al. Meta-analysis of the effects of helmet-assisted non-invasive ventilation in the treatment of acute respiratory failure. Eur Rev Med Pharmacol Sci. 2019;23(10):4382–90. 10.26355/eurrev_201905_17945 31173312

[29] Mayordomo-Colunga J, Rey C, Medina A, Martinez-Camblor P, Vivanco-Allende A, Concha A. Helmet Versus Nasal-Prong CPAP in Infants With Acute Bronchiolitis. Respir Care. 2018;63(4):455–63. 10.4187/respcare.05840 29382794

[30] Chidini G, Piastra M, Marchesi T, De Luca D, Napolitano L, Salvo I, et al. Continuous positive airway pressure with helmet versus mask in infants with bronchiolitis: an RCT. Pediatrics. 2015;135(4):e868–75. 10.1542/peds.2014-1142 25780074

[31] Chidini G, Calderini E, Cesana BM, Gandini C, Prandi E, Pelosi P. Noninvasive continuous positive airway pressure in acute respiratory failure: helmet versus facial mask. Pediatrics. 2010;126(2):e330–6. 10.1542/peds.2009-3357 20660548

[32] Patroniti N, Foti G, Manfio A, Coppo A, Bellani G, Pesenti A. Head helmet versus face mask for non-invasive continuous positive airway pressure: a physiological study. Intensive Care Med. 2003;29(10):1680–7. 10.1007/s00134-003-1931-8 14564379

[33] Vaschetto R, De Jong A, Conseil M, Galia F, Mahul M, Coisel Y, et al. Comparative evaluation of three interfaces for non-invasive ventilation: a randomized cross-over design physiologic study on healthy volunteers. Crit Care. 2014;18(1):R2. 10.1186/cc13175 24387642 PMC4056758

[34] Meira C, Joerger FB, Kutter A, Waldmann A, Ringer SK, Boehm SH, et al. Comparison of three continuous positive airway pressure (CPAP) interfaces in healthy Beagle dogs during medetomidine-propofol constant rate infusions. Vet Anaesth Analg. 2018;45(2):145–57. 10.1016/j.vaa.2017.11.001 29422335

[35] Marshall H. Non-invasive ventilation by helmet more effective than face mask in acute respiratory distress syndrome. Lancet Respir Med. 2016;4(8):610. 10.1016/S2213-2600(16)30183-7 27373812

[36] Ali A, Turkmen A, Turgut N, Altan A, Sari T. [Comparison of non-invasive mechanical ventilation with helmet or face mask in patients with acute exacerbation of chronic obstructive pulmonary disease]. Tuberk Toraks. 2011;59(2):146–52. 10.5578/tt.738 21740389

[37] Antonaglia V, Ferluga M, Molino R, Lucangelo U, Peratoner A, Roman-Pognuz E, et al. Comparison of noninvasive ventilation by sequential use of mask and helmet versus mask in acute exacerbation of chronic obstructive pulmonary disease: a preliminary study. Respiration. 2011;82(2):148–54. 10.1159/000324259 21447934

[38] Pisani L, Mega C, Vaschetto R, Bellone A, Scala R, Cosentini R, et al. Oronasal mask versus helmet in acute hypercapnic respiratory failure. Eur Respir J. 2015;45(3):691–9. 10.1183/09031936.00053814 25504992

[39] Zhang W, Shen C. The clinical use of noninvasive positive pressure ventilation using a helmet in patients with acute hypoxaemic respiratory failure. Journal of Clinical Pulmonary Medicine. 2010(1):48–50.

[40] Wang Z. Effect of different interfaces of non-invasive positive pressure ventilation on hypoxemia after coronary artery bypass grafting. Journal of Clinical Medicine in Practice. 2017;21(17):99–102, 105.

[41] Yang Y, Liu N, Hou X, Sun L, Wang H. Effects of noninvasive positive pressure ventilation with different interfaces on hypoxemic patients after coronary artery bypass grafting surgery. Journal of Cardiovascular and Pulmonary Diseases. 2016(2):110–5.

[42] Zhang W, Shen C. Clinical use of noninvasive positive pressure ventilation using a helmet in patients with acute exacerbation of chronic obstructive pulmonary disease. International Journal of Respiration. 2008(15):900–3.

[43] Ma F, Zhang Z, Li B, Yue W, Hou Y, Zhang C. Clinical Effect of Noninvasive Positive Pressure Ventilation for Chronic Obstructive Pulmonary Disease. Chinese Journal of Medical Guide. 2019;21(1):7–11.

[44] BaHammam AS, Singh TD, Gupta R, Pandi-Perumal SR. Choosing the Proper Interface for Positive Airway Pressure Therapy in Subjects With Acute Respiratory Failure. Respir Care. 2018;63(2):227–37. 10.4187/respcare.05787 29089459

[45] Chidini G, Piastra M, Marchesi T, De Luca D, Napolitano L, Salvo I, et al. Continuous positive airway pressure with helmet versus mask in infants with bronchiolitis: an RCT. Pediatrics. 2015;135(4):e868–75. 10.1542/peds.2014-1142 25780074

[46] Taccone P, Hess D, Caironi P, Bigatello LM. Continuous positive airway pressure delivered with a "helmet": effects on carbon dioxide rebreathing. Crit Care Med. 2004;32(10):2090–6. 10.1097/01.ccm.0000142577.63316.c0 15483419

[47] Antonelli M, Pennisi MA, Pelosi P, Gregoretti C, Squadrone V, Rocco M, et al. Noninvasive positive pressure ventilation using a helmet in patients with acute exacerbation of chronic obstructive pulmonary disease: a feasibility study. Anesthesiology. 2004;100(1):16–24. 10.1097/00000542-200401000-00007 14695719

[48] Patel BK, Wolfe KS, MacKenzie EL, Salem D, Esbrook CL, Pawlik AJ, et al. One-Year Outcomes in Patients With Acute Respiratory Distress Syndrome Enrolled in a Randomized Clinical Trial of Helmet Versus Facemask Noninvasive Ventilation. Crit Care Med. 2018;46(7):1078–84. 10.1097/CCM.0000000000003124 29595563 PMC6005726

[49] Kyeremanteng K, Gagnon LP, Robidoux R, Thavorn K, Chaudhuri D, Kobewka D, et al. Cost Analysis of Noninvasive Helmet Ventilation Compared with Use of Noninvasive Face Mask in ARDS. Can Respir J. 2018;2018:6518572. 10.1155/2018/6518572 29670676 PMC5833880

